# Editorial: Recent advances in molecular and structural endocrinology

**DOI:** 10.3389/fendo.2023.1178265

**Published:** 2023-03-23

**Authors:** Pierre De Meyts, Stefan N. Constantinescu

**Affiliations:** ^1^ Cell Signalling Pole, de Duve Institute, Université catholique de Louvain, Brussels, Belgium; ^2^ Ludwig Cancer Institute, Brussels, Belgium; ^3^ Walloon Excellence in Life Sciences and Biotechnology, Brussels, Belgium; ^4^ Nuffield Department of Medicine, Ludwig Institute for Cancer Research, Oxford University, Oxford, United Kingdom

**Keywords:** editorial, recent advances, molecular endocrinology, structural endocrinology, research topic

Frontiers in Endocrinology has organized a series of Research Topics to highlight the latest advances in research across the field of Endocrinology, with articles from the Associate Members of our Editorial Boards. This editorial initiative was focused on new insights, novel developments, current challenges, latest discoveries, recent advances, and future perspectives in the field. This Research Topic for the section on Molecular and Structural Endocrinology was edited by Specialty Chief Editor Pierre De Meyts and Associate Editor Stefan Constantinescu, who are grateful to the Associate Editors who submitted a contribution, as well as to an outside submission.

Two papers address the structure-function relationships of the insulin molecule. Insulin exerts its metabolic and mitogenic effects through binding to the insulin receptor, a member of the receptor tyrosine kinase family, as well as weak binding to the insulin-like growth factor-1 (IGF-1) receptor In this collection, the review by Gorai and Vashisth highlights the contributions of molecular dynamics (MD) simulations in elucidating the atomic-level details of conformational dynamics in the insulin molecule, where the structure of the hormone has been probed as a monomer, dimer and hexamer. They provide a comprehensive analysis of 81 articles published since 1985 (grouped by 5-year periods, stressing the methodological improvements) that have implemented molecular modeling techniques to study insulin structure, including the effect of solvent, pH, temperature and pressure at the microscopic scale. They highlight the conformational dynamics underlying the activities of insulin analogues and mimetics. They also briefly discuss the future prospects for computational methods in developing promising synthetic insulin analogues. The second paper on insulin by Ong et al. addresses the way in which specific agonists of the insulin receptor lead to disproportionate metabolic or mitogenic actions, so-called “biased” signaling, the mechanism of which is poorly understood. The best-known example is the supermitogenic HisB10Asp mutated insulin analogue. Here the authors report a novel insulin analogue with an opposite type of biased signaling, having low mitogenic potency. The authors changed the chemical composition of the A6-A11 disulfide bond to a rigid, non-reducible C=C linkage (*cis-*dicarba bond) in both native insulin and the basal/long-acting analogue insulin glargine. Detailed biochemical experiments showed that the reduced mitogenicity appears to be due to a poor ability to induce insulin receptor internalization with resulting poor activation of the ERK1/2 signaling pathway. The insight generated by this kind of study could ultimately help designing safer insulin analogues with low mitogenicity for the treatment of diabetes.

Two original companion research articles from Chen et al. (Chen et al. and Racca et al.) are focused on understanding genotype-phenotype relationships in mutants of the therian mammals’ Y-encoded SRY transcription factor, that is responsible for male sex determination. SRY contains a high-mobility-group (HMG) box mediating sequence-specific DNA bending, an important process regulating DNA function. Mutations clustering in this box cause XY gonadal dysgenesis (Swyer syndrome) and usually arise *de novo*, but are also rarely inherited. In these papers, the authors tested two *de novo* mutations (Y127H and Y127C) and one inherited mutation (Y127F). Y127H and Y127C reduced SRY activity in SRY-responsive cell lines by 5- and 7-fold, respectively. Whereas Y127H impaired testis-specific enhancer activity, Y127C accelerated proteasomal proteolysis. The inherited Y127F variant was better tolerated, and its activity reduced only twofold. Variant HMG boxes had similar DNA affinities and showed only subtle differences in sharp DNA bending. Such modest perturbations are within the range of species variation. Tyr and Phe differ only by the presence or absence of a *para*-hydroxyl group in corresponding aromatic rings, a subtle chemical modification of a transcription factor nevertheless underlying a dramatic phenotypic outcome. The authors propose that the *para*-hydroxyl group of Y127 enhances the gene-regulatory activity of SRY, and that Y127 serves as an *anchor point* for a bound water molecule bridging the protein and DNA interfaces. This bound water molecule is envisioned to lock the bent protein-DNA complex to prolong its lifetime and enhance the precision of DNA bending. Loss of the Y127 anchor among SRY variants presumably unclamps its basic tail, causing a rapid DNA dissociation despite native affinity and attenuated transcriptional activity at the edge of sexual ambiguity.

The *O*-linked β-N-Acetylglucosamine (*O-*GlcNAc) regulatory system has been a popular topic in this journal. It is traditionally considered a glucose metabolism-associated protein modification. The comprehensive review (302 references) by Lockridge and Hanover describes how this system interacts extensively and bi-directionally with lipids and is required to maintain lipid homeostasis. *O-*GlcNAc cycling enzymes act as broad-spectrum environmental sensors and provide both acute and long-term adaptation to stress and other environmental stimuli such as nutrient availability. Hyperlipidemia modulates *O-*GlcNAc levels by targeting UDP-GlcNAc synthesis and the glycosylation enzymes *O-*GlcNAc transferase (OGT) and *O-*GlcNAcase (OGA). Reciprocally, OGT activity homeostatically regulates systemic lipid uptake, storage and release. We cannot summarize here the numerous interconnections between lipid and *O-*GlcNAc metabolism examined in this review, which provides insights into how the *O-*GlcNAc regulatory system may contribute to lipid-associated diseases including obesity and the metabolic syndrome.

Orexins also called hypocretins were simultaneously discovered in 1998 by two independent groups. The review by Couvineau et al. focuses on structural and anti-tumoral aspects of orexins and their receptors. Orexin-A/hypocretin-1 and orexin-B/hypocretin-2 are two neuropeptides expressed in the hypothalamus as a prepro-orexins precursor. They interact with two G protein-coupled receptor isoforms called OX1R and OX2R. They play an important role in the central and peripheral nervous systems where they control multiple processes: wakefulness, addiction, reward seeking, stress, motivation, memory, energy homeostasis, food intake, blood pressure, hormone secretions, reproduction, gut motility and lipolysis. They are involved in multiple pathologies including narcolepsy type 1, inflammation, neurodegenerative diseases, metabolic syndrome and cancers. OX1R is expressed in digestive cancers encompassing colon, pancreas and liver cancer. A wide range of pharmacological molecules have been designed for structural studies. Recent studies have demonstrated an anti-inflammatory and anti-tumoral action of orexins. This field appears to be ripe for further development in human health.

Insulin-stimulated glucose uptake in skeletal muscle is an essential mechanism that prevents postprandial hyperglycemia and is responsible for 80% of the peripheral glucose uptake from the circulation *via* the insulin-responsive glucose transporter GLUT4. In the basal state, GLUT4 is sequestered in intracellular storage vesicles. In response to insulin, the GLUT4 storage vesicles rapidly translocate to the plasma membrane where they undergo vesicle docking, priming and fusion. This process is governed by high affinity interactions among the SNARE (soluble N-ethylmaleimide sensitive factor attachment protein receptor) exocytosis proteins and their regulators. Defects in GLUT4 translocations and in several SNARE proteins and their regulators have been linked to insulin resistance and type 2 diabetes. A comprehensive review (237 references) by Hwang and Thurmond highlights the latest research on the role of SNAREs and their regulatory proteins in insulin-stimulated GLUT4 translocation in skeletal muscle. They also discuss novel emerging roles of SNARE proteins as interaction partners in pathways not usually thought to involve SNARES, possibly unraveling novel therapeutic targets for treating insulin resistance and type 2 diabetes.

In the last paper, an original research article, Di Vincenzo et al. develop a promising *in vitro* model to study Cushing’s syndrome. They use undifferentiated, self-renewing progenitors of adipocytes, mesenchymal stem cells (MSCs) from the abdominal skin of healthy subjects and treat them thrice daily with glucocorticoids according to two different regimens: lower, circadian-decreasing exposure *versus* persistently higher doses, mimicking either the physiological condition or Cushing’s syndrome, respectively. They found that lower decreasing dosing did not impair glucose uptake by MSCs, while persistently high dosing decreased it, resulting in insulin resistance after 30 hours. In the latter condition, lipolysis-related genes were acutely downregulated followed by overexpression once insulin resistance was established.

This interesting selection of highlights in no way pretends to represent the whole spectrum of recent advances in the field of Molecular and Structural Endocrinology. There has been considerable progress recently in solving the three-dimensional structure of the receptors for insulin and IGF-1 receptors in their apo- and liganded forms due to improvements in the resolution power of cryo-electron microscopy for review, see ref. (1). This will be the topic of an upcoming Research Topic in this journal. Progress in this field may open the door to the development of new approaches in diabetes management.

## Author contributions

PM wrote the editorial, SC read, corrected, and approved it.

## References

[1] Forbes BE. The three-dimensional structure of insulin and its receptor. Vitamins Hormones (2023). 10.1016/bs.vh.2022.12.001 37717984

